# *Daphnia galeata* responds to the exposure to an ichthyosporean gut parasite by down-regulation of immunity and lipid metabolism

**DOI:** 10.1186/s12864-018-5312-7

**Published:** 2018-12-14

**Authors:** Yameng Lu, Paul R. Johnston, Stuart R. Dennis, Michael T. Monaghan, Uwe John, Piet Spaak, Justyna Wolinska

**Affiliations:** 1 Leibniz Institute of Freshwater Ecology and Inland Fisheries (IGB), Berlin, Germany; 2 Berlin Center for Genomics in Biodiversity Research (BeGenDiv), Berlin, Germany; 3 Freie Universität Berlin, Berlin, Germany; 4 Swiss Federal Institute of Aquatic Science and Technology (Eawag), Dübendorf, Switzerland; 5 Alfred Wegener Institut Helmholtz Zentrum für Polar und Meeresforschung (AWI), Bremerhaven, Germany; 6 Helmholtz Institute for Functional Marine Biodiversity (HIFMB), Oldenburg, Germany

**Keywords:** *Daphnia*, Differential expression, Host-parasite interaction, Immune response, RNAseq

## Abstract

**Background:**

Regulatory circuits of infection in the emerging experimental model system, water flea *Daphnia* and their microparasites, remain largely unknown. Here we provide the first molecular insights into the response of *Daphnia galeata* to its highly virulent and common parasite *Caullerya mesnili*, an ichthyosporean that infects the gut epithelium. We generated a transcriptomic dataset using RNAseq from *parasite-exposed* (vs. *control*) *Daphnia*, at two time points (4 and 48 h) after parasite exposure.

**Results:**

We found a down-regulation of metabolism and immunity-related genes, at 48 h (but not 4 h) after parasite exposure. These genes are involved in lipid metabolism and fatty acid biosynthesis, as well as microbe recognition (e.g. c-type lectins) and pathogen attack (e.g. gut chitin).

**Conclusions:**

General metabolic suppression implies host energy shift from reproduction to survival, which is in agreement with the known drastic reduction in *Daphnia* fecundity after *Caullerya* infection. The down-regulation of gut chitin indicates a possible interaction between the peritrophic matrix and the evading host immune system. Our study provides the first description of host transcriptional responses in this very promising host-parasite experimental system.

**Electronic supplementary material:**

The online version of this article (10.1186/s12864-018-5312-7) contains supplementary material, which is available to authorized users.

## Introduction

Ecological and evolutionary outcomes of host-parasite interactions are receiving renewed attention [1, 2]. It remains challenging to identify model host-parasite systems that are abundant in nature and amenable to experimental manipulation in the laboratory. Such systems are necessary to better understand the mechanistic basis determining the outcome of host-parasite encounters, such as immune response or general metabolic responses of the host. Zooplankton water fleas *Daphnia* (Crustacea: Cladocera) and their microparasites comprise a promising model system that fulfills these requirements. *Daphnia* are abundant in standing freshwater bodies and a key component of aquatic food webs as grazers of phytoplankton and a major food source for planktivorous fish and some invertebrates [3]. *Daphnia* are often infected by a variety of microparasites in nature, including bacteria, fungi, microsporidia and protozoans [4]. Some of these parasites are highly virulent, inducing a strong reduction in host fitness [4, 5] and affecting the outcome of competition between host genotypes [6, 7] or species [8, 9]. *Daphnia* are easy to maintain in laboratory cultures in conditions where females reproduce asexually via parthenogenesis [4, 10]. Their parasites can also be cultured in vivo in the lab [4, 11].

In the last two decades, *Daphnia* have been used to study host-parasite interactions [4, 11], becoming one of the 13 model organisms for biomedical research (https://grants.nih.gov/grants/policy/model_organism/). *Daphnia* have been also used for investigating the evolution of immunity [12, 13], facilitated by an increasing availability of genomic information [14–16]. The immune system of arthropods is well described, especially for laboratory model species such as fruit flies *Drosophila* [17, 18] and mealworm beetles *Tenebrio* [19], or for vectors of human diseases such as mosquitoes [20]. Some immunity pathways of arthropods are highly conserved between insects and *Daphnia* [12]. In *Daphnia pulex,* adaptive evolution in immune system genes is stronger than in non-immune genes [13], as it has also been observed across different *Drosophila* species under a broad gradient of selective pressures [21]. However, the response of *Daphnia* immune-related genes to individual parasites is not yet well understood.

Only two studies have assessed the transcriptomic response of *Daphnia* to parasites, and both used *D. magna* exposed to the bacterium *Pasteuria ramosa.* The first study found 45 differentially expressed (DE) genes at 96 h (but not at 40 or 144 h) after parasite exposure; these DE genes were not enriched for Gene Ontology (GO) terms [22]. In the second study, a single immune-related gene, inducible nitric oxide synthase (iNOS), was down-regulated at 4 h (but not at 8 or 12 h) after parasite exposure [23]. The host species in these studies, *D. magna*, inhabits small, fishless water bodies. Permanent lakes and reservoirs, however, are inhabited by smaller *Daphnia* species that can withstand fish predation, such as *D. galeata* [24–26]. One of the most abundant parasites of *Daphnia* species inhabiting European lakes and reservoirs is the gut parasite *Caullerya mesnili* (Protista: Ichthyosporeans) [27]. *C. mesnili* causes regular epidemics with prevalence of up to 40% of the entire *Daphnia* population [6, 28, 29]. *C. mesnili* can be propagated in vivo in laboratory *Daphnia* cultures. It spreads horizontally from infected to uninfected *Daphnia* and a new infection is visible from 8 to 12 days after parasite exposure [30]. Infected *Daphnia* suffer highly reduced fecundity and survival [27, 31]. Despite the crucial role of *C. mesnili* in the maintenance of host species [8] and genetic [6] diversity, the mechanisms of how *C. mesnili* induces host’s defense/adaptive responses to the parasite are unknown.

Here we use Illumina MiSeq RNA-sequencing to produce a transcriptome-wide profile of the response of *D. galeata* following exposure to the gut parasite *C. mesnili*, at 4 and 48 h after parasite encounter. We aimed to understand how different components of the *Daphnia* immune system are recruited and how general metabolic pathways are regulated at a transcriptomic level. We also improved annotation of the *D. galeata* transcriptome by identifying specific orthologs of immune-related genes to facilitate further studies*.*

## Materials and methods

### Host-parasite cultures

Two parasite strains were used in the experiment: i) *C. mesnili* strain isolated from lake Greifensee (Switzerland) and ii) *C. mesnili* strain isolated from lake Skulska Wies (Poland). Both strains were isolated in September 2016 (7 months before the experiment was conducted) and separately maintained on a single *D. galeata* host clone (G100; standard laboratory clone, inbred once) by adding newborns from uninfected stock cultures at approximately 2-week intervals [8]. Both uninfected and infected *D. galeata* cultures were kept in synthetic *Daphnia* medium [32], at a constant temperature of 20 ± 1 °C, on a light: dark cycle of 12 h:12 h and with an unlimited food supply of the unicellular green algae *Scenedesmus obliquus* (> 1 mgL^− 1^ C, added three times per week).

### Experimental setup

We aimed to determine the transcriptional changes of the host *D. galeata* in response to parasite *C. mesnili* exposure at two harvesting time points post-exposure (4 and 48 h). To minimise maternal effects, the *Daphnia* (G100 clone) were cultured for two generations under standardized conditions as described above, except that they were fed daily with 1 mgL^− 1^ C *S. obliquus*, and their age and densities were controlled; 10 individuals were raised per jar in 200 mL medium. Adult females (between 21 and 30 days old) were transferred from these stock cultures into six experimental jars containing 200 mL medium each. We standardised the reproductive status of experimental populations, by using 26 gravid and 19 non-gravid females individuals (without eggs or developed ovaries, as checked under a microscope) per jar. *Daphnia* were fed with 0.5 mgL^− 1^ C *S. obliquus*. On the next day, neonates were removed and dead adults were replaced, if necessary. Then, *parasite-homogenate* and *control-homogenate* were added (each to three jars). *Parasite-homogenate* was prepared from G100-individuals heavily infected with *C. mesnili.* The infected individuals were obtained from stock parasite cultures (34 individuals infected with Greifensee strain and 10 individuals infected with Skulska Wies strain, there was a lower availability of the later strain in laboratory cultures). This or lower parasite/host ratio (i.e. ~ 1 donor *Daphnia* / 3 recipient *Daphnia*) has been proven to result in successful infections [8, 33, 34]. Infected *Daphnia* were homogenized in an Eppendorf tube, using a pestle. The homogenate as well as the medium used to maintain infected *Daphnia* (infected individuals were selected from stock cultures 24 h beforehand) was equally distributed across the three replicates of *parasite-exposed* treatment. *Control-homogenate* was similarly prepared using 44 uninfected hosts and distributed across three replicates of *control* treatment. After 4 h and then after 48 h post exposure, 11 to 20 *Daphnia* individuals were collected from each experimental unit. *Daphnia* were homogenized in an Eppendorf tube with a disposable plastic pestle by hand (~ 5 min), in 150 μl TRIzol reagent (Invitrogen, Carlsbad, USA) at room temperature. A further 850 μl TRIzol reagent was added and the sample was shaken vigorously by hand for 15 s, before being quenched in liquid nitrogen and frozen at − 80 °C.

### RNA preparation, library construction and sequencing

Total RNA was extracted from *parasite-exposed* and *control* treatments (3 replicates each) at each time point (4 and 48 h post-exposure). 200 μl chloroform was added to each homogenate after thawing. Each sample was shaken vigorously by hand, incubated for 2–3 min at room temperature and then centrifuged at 13,000 g for 15 min at 4 °C. The upper aqueous phase (approx. 540 μl) was transferred to a new RNase-free 1.5 ml microcentrifuge tube and 0.5x volume of absolute ethanol was added to precipitate nucleic acids. The tubes were mixed by inverting 10 times. RNA purification, including on-column DNA digestion, was performed using the RNeasy Kit (Qiagen, Hilden, Germany), according to the manufacturer’s protocol. The quality and quantity of the RNA were determined using a NanoDrop ND-1000 spectrometer (PeqLab, Erlangen, Germany) and an RNA Nano Chip assay on a Bioanalyzer 2100 device (Agilent Technologies, Böblingen, Germany).

Libraries were prepared using the TruSeq Stranded mRNA HT Library Preparation Kit (Illumina, San Diego, USA), according to the manufacturer’s protocol. The libraries were quality checked and quantified using a Bioanalyzer 2100 and a DNA Chip assay. Sequencing was performed on a NextSeq 500 instrument (Illumina, San Diego, USA) as 150-bp paired-end Illumina library. Sequence information was extracted using the CASAVA software (Illumina, San Diego, USA) in FASTQ format. The analysis produced 12 data sets for paired-end sequencing.

### Reference transcriptomes

Salmon version 0.8.2 [35] with default parameters was used to pseudo-align raw RNAseq reads to a *published D. galeata* transcriptome containing 32,903 *D. galeata* contigs from a mixture of 24 clones isolated from four different lakes [36]. The quality control of this transcriptome was done by Huylmans et al. [36], including the extraction of the longest ORF, the comparison with single copy genes and a MEGAN approach for the presence of non-*Daphnia* transcripts. To ensure no relevant transcripts were overlooked, a de novo transcriptome was created using RNAseq data produced in this study, resulting in 370,978 contigs (from a single *D. galeata* clone G100). The de novo transcriptome was assembled using Trinity version 2.4.0 [37] with default parameters. This included quality and adapter trimming using trimmomatic version 0.36 [38] (ILLUMINACLIP:TruSeq3-PE.fa:2:30:10 SLIDINGWINDOW:4:5 LEADING:5 TRAILING:5 MINLEN:25) followed by in silico normalization to a maximum coverage of 50. The de novo transcriptome was functionally annotated following the trinotate annotation suite guidelines (https://trinotate.github.io).

### Analyses of RNAseq data

After pseudo-alignment of the raw reads with Salmon [35], differentially expressed (DE) transcripts between *parasite-exposed* and *control Daphnia* were determined for each time point. This was done for *published* and de novo reference transcriptome separately, using the R [39] package DESeq2 [40] in conjunction with tximport [41] to aggregate transcript abundances at the gene level. Dispersions were modeled using a local fit with discovery rates at Benjamini-Hochberg-adjusted *P*-value < 0.05 and absolute value of log2FoldChange > 1. All DE transcripts were translated into amino acid sequences using the Virtual Ribosome package [42] and assigned to eukaryotic orthologous groups (KOGs) with an e-value cut-off of 1e^− 5^ using the Batch Web CD search tool [43]. Putative orthologs were predicted from reciprocal best BLAST hits. Protein families and signal peptides were identified using Pfam [44] and SignalP [45], respectively. The annotated Pfam domains were transferred to Gene Ontology (GO) terms with Pfam2GO [46].

Only these transcripts that were identified from the *published* transcriptome were used for GO enrichment analysis. This is because the *published* transcriptome was quality controlled so redundant contigs were removed [36], whereas the de novo assembly primarily created duplicated contigs. Such redundancies may affect the enrichment analysis as several contigs derived from the same gene would be incorrectly counted. In order to identify over-represented functions (significant at *P*-value < 0.05) among DE genes, GO enrichment analysis was performed with a hypergeometric distribution using R package GOstats tool [47].

Annotated transcripts (*published* reference transcriptome) were grouped into manually curated categories based on literature searches highlighting immune functions and based on the information in a *Daphnia* immunity database [12].

## Results

### Differentially expressed genes

**Fig. 1:**
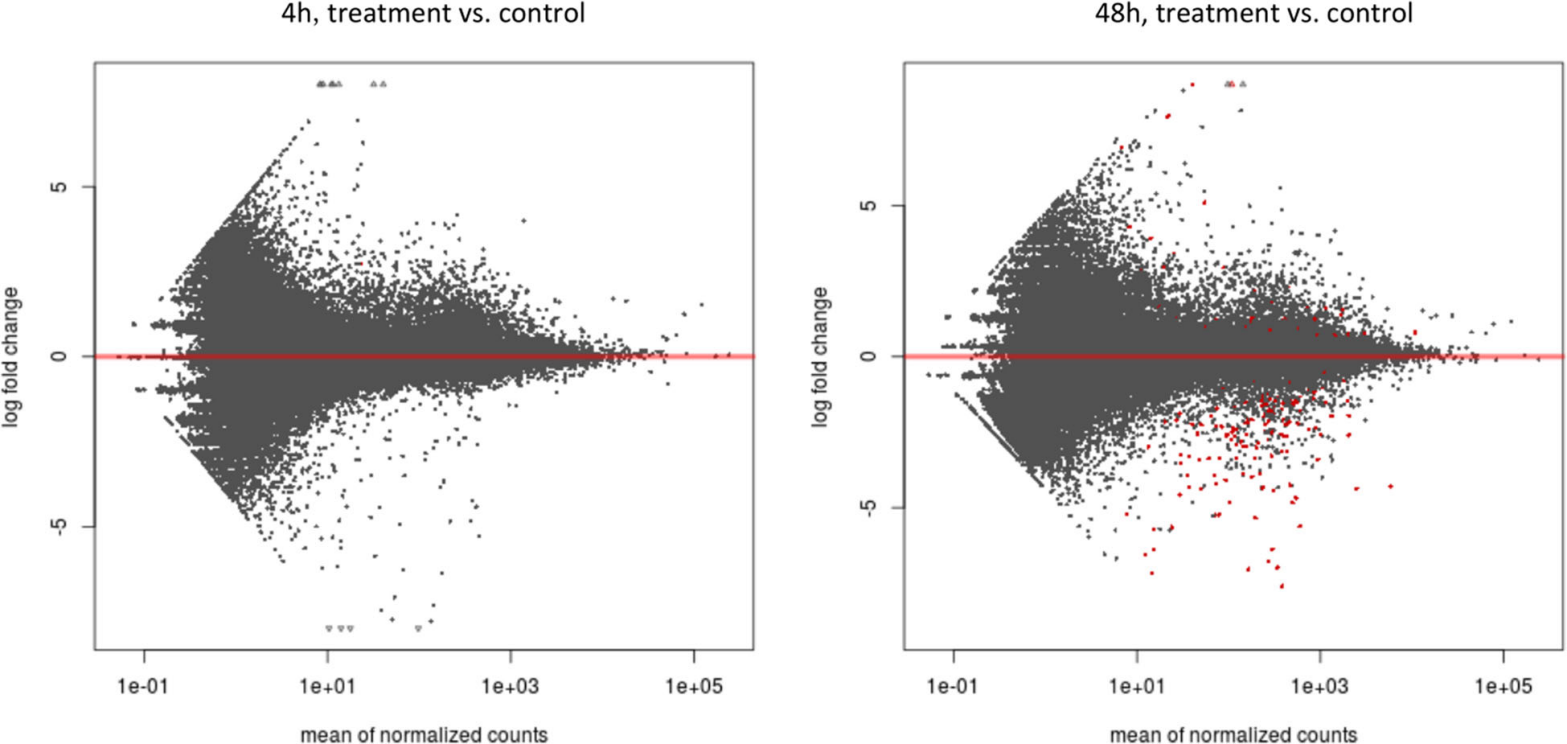
Volcano plots displaying differential gene expression in *Daphnia galeata* after exposure to parasite at 4 (left) and 48 (right) hours. Each point represents an individual gene transcript. Red points represent significantly differentially expressed transcripts (Benjamini-Hochberg-adjusted *P*-value < 0.05)

**Fig. 2:**
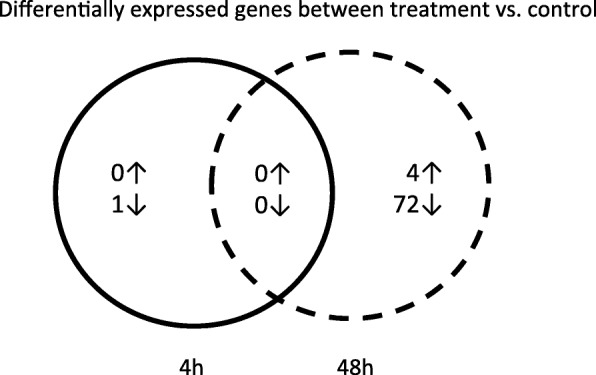
Venn diagram of the differentially up-regulated (↑) and down-regulated (↓) gene expression in *Daphnia galeata* at 4 h (solid circle) and 48 h (dashed circle) after parasite exposure. There was no overlap of differentially expressed genes between two time points. Numbers presented here are based on the *published* reference transcriptome [36], for a similar venn diagram based on the de novo reference transcriptome see Additional file 2: Figure S1

There were more significantly differentially expressed (DE) genes, as assessed for *parasite-exposed* vs. *control* samples, at 48 h than at 4 h post exposure (Fig. 1; the full list of DE genes for each treatment, their accession numbers, *P*-values and log_2_FC values are provided in Additional file 1: Table S1). Specifically, after 4 h one gene was down-regulated (detected against the *published* reference transcriptome), and one up-regulated (detected only against the de novo transcriptome). After 48 h, there were 72 (137) down-regulated and four (31) up-regulated genes, according to the *published* (Fig. 2) or, in parentheses, de novo transcriptome (Additional file 2: Figure S1). There was no overlap of DE genes between two time points (4 and 48 h).

### Functional categorization of DE genes

All DE genes were annotated (Additional file 3: Table S2). At the early time point after exposure (4 h), the only up-regulated gene had a putative function of heat shock protein 40 (*Hsp40*, *E-*value = 1.45E-15), whereas the only down-regulated gene was not assigned to any putative function (see Additional file 3: Table S2).

**Fig. 3:**
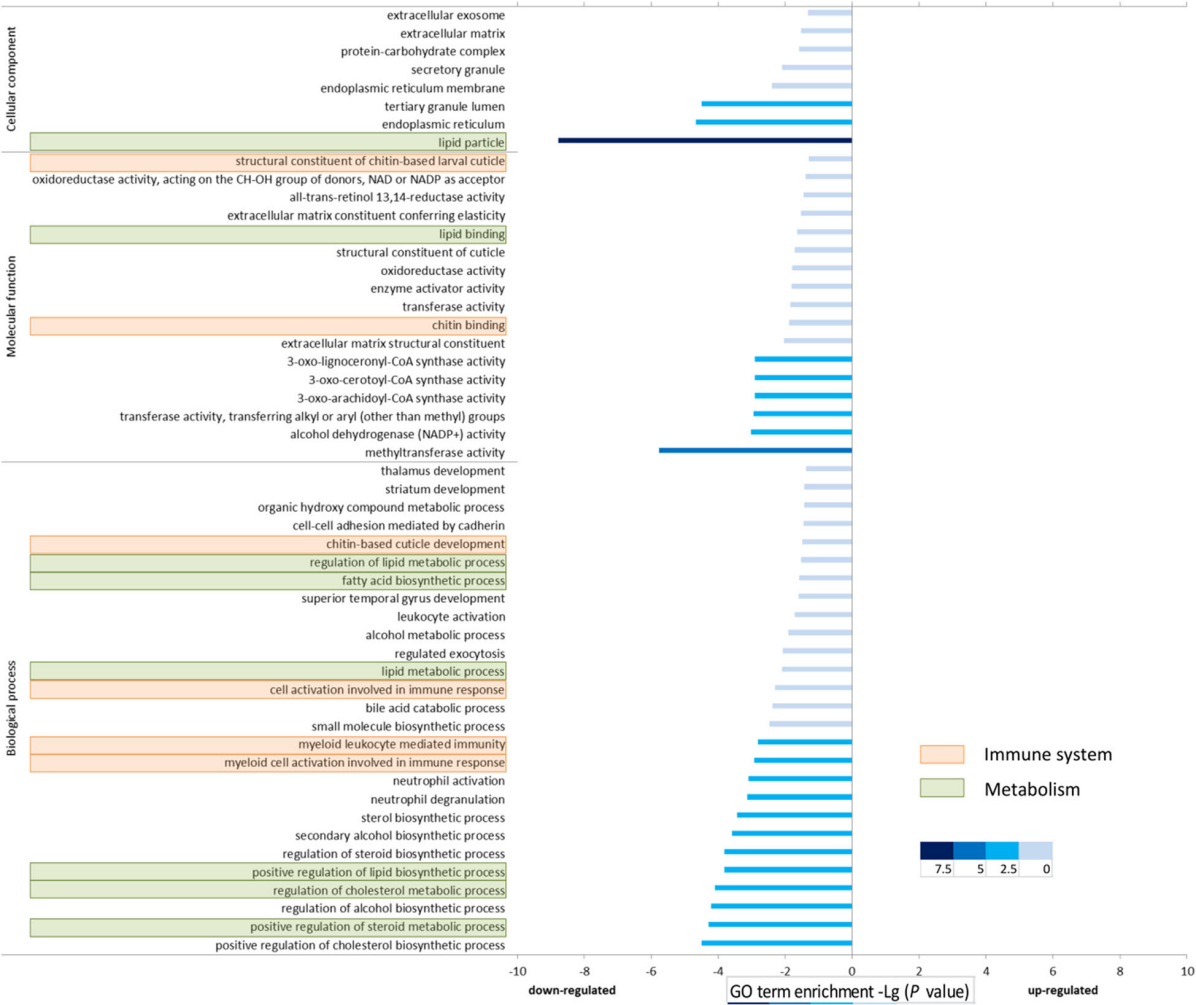
Gene Ontology (GO) [79] enrichment distributions of *Daphnia galeata* genes that were down-regulated in *parasite-exposed* treatment compared to the *control* treatment at 48 h after parasite exposure. No enriched GO term was identified for the up-regulated genes. Two functional categories are highlighted: metabolism- (green) and immune- (orange) related GO terms

The DE genes identified based on the *published* transcriptome were used for Gene Ontology (GO) enrichment analysis. The DE genes were enriched for 52 GO terms. These GO terms were classified within three ontologies: cellular component, molecular function, and biological process. After 48 h, eight enriched GO terms were related to lipid metabolism or biosynthesis, and six enriched GO terms were associated with immunity pathways (Fig. 3 and Additional file 4: Table S3).

### Immune-related genes

Immune-related genes were defined as transcripts encoding putative orthologs or predicted proteins of *Daphnia* immune system. There were 82 transcripts in the immune protein families that were expressed in the *published* reference transcriptome. These transcripts were grouped into four main functional classes: pathogen recognition, signal transduction, attack and others (Table 1). A full list of the immune-related genes is provided in Additional file 5: Table S4. Several immune-related genes were down-regulated at 48 h after parasite exposure, such as c-type lectins related to microbe recognition and different chitinases involved in the defense against pathogen attack. Only one immune-related gene, Cytoglobin (Cygb) related to nitric oxide synthase (iNOS) and oxidative stress, was up-regulated at 48 h. At the early stage (4 h), exposure to parasites did not cause a significant change in the expression of immune-related genes (Table 2).

**Table 1:** Annotated gene copy number across different immunity pathways as identified from a *published* transcriptome of *Daphnia galeata* (data set containing 32,903 contigs) [36]. Transcripts are grouped into four main functional classes

Functional category	Protein family	Number of transcripts
Recognition	TEP (thioester containing proteins)	7
	GNBP (gram-negative bingding proteins)	11
	Scavenger	6
	C-type lectin	6
	Galetin	3
Transduction	Toll	7
	MyD88	1
	Pelle	1
	Relish	1
	Imd	1
	Cactus	1
	STAT (signal transducer and activator of transcription)	1
Attack	Chitinase	17
	Prophenoloxidase	1
	Caspase	8
	Nitric oxide synthase	2
Others	Argonaute	2
	Dicer	3
	DSCAM (down syndrome cell adhesion molecule)	1
	Dorsal	1
	Gemini	1
Total number		82

**Table 2:** Immune-related genes in *Daphnia galeata* that were up- (↑) or down-regulated (↓) at 48 h after parasite exposure (based on *published* reference transcriptome [36])

Functional category	Different expression			Functional annotation			
	Regulation	P-adj	Log2FoldChange	Pfam family name	E-value	Accession	Contig name
Recognition	↓	0.0008	−5.1719	Lectin_C	0.0012	pfam00059	Dgal_o2484d46587t1
Attack	↓	3.66E-05	−4.1466	CBM_14	1.81E-06	pfam01607	Dgal_o12557t2
	↓	1.45E-05	−5.2366	Chitin_bind_4	0.0004	pfam00379	Dgal_t22909c0t1
	↓	5.83E-04	−2.3732	Chitin_bind_4	0.0011	pfam00379	Dgal_o2545d42932t1
	↓	1.03E-03	−5.2741	Cuticle_3	0.0053	pfam11018	Dgal_t23153c1t3
	↓	1.42E-03	−2.2140	Chitin_bind_4	3.46E-09	pfam00379	Dgal_a24_b_768727
	↓	2.16E-03	−2.3542	Cuticle_3	0.0053	pfam11018	Dgal_o6t1664
	↑	0.0137	7.3712	Cygb	2.81E-12	cd08924	Dgal_o503t5
	↓	2.28E-02	−1.5097	E_set superfamily	1.81E-23	cl09101	Dgal_t24657c0t1
	↓	0.0469	−1.1718	Glyco_hydro_18	3.25E-67	pfam00704	Dgal_s418763

## Discussion

The results presented here indicate the existence of coordinated feedbacks occurring between metabolic and immunity pathways in *Daphnia galeata* in response to *Caullerya mesnili* infection. This was revealed by changes in gene expression after parasite exposure compared to the uninfected controls. Soon after parasite exposure (4 h), there were very few differentially expressed (DE) genes compared to after 48 h. Only a stress response was triggered in *Daphnia* as an early action against *C. mesnili*, whereas the later response resulted in an immune and general metabolic suppression.

### Early response

At the early stage (4 h) only a single gene, heat shock protein 40 (*Hsp40*), was up-regulated. Heat shock proteins are highly conserved proteins and indicators of initial response to different environmental stressors in prokaryotic and eukaryotic organisms [48–50]. The changes in the level of heat shock proteins in *Daphnia* are induced under thermal stress [51], as a reaction to predatory cues [52] or different pollutants [53, 54]. Moreover, an increase of *Hsp60* levels was observed in a natural population of *Daphnia magna* infected by the ectoparasite *Amoebidium parasiticum* [55]. Apart from the up-regulation of *Hsp40* and the down-regulation of one gene not assigned to any putative function, there were no other differentially regulated genes at the early stage after parasite exposure. It might be that the level of threat from the parasite was still too low to trigger the response of the host, or that the host had not yet mounted an immune response specific to *C. mesnili* attack. A microarray study of *D. magna* exposed to bacterial endoparasite *Pasteuria ramosa* showed even more delayed response. In that study, 45 genes were differentially expressed 96 h after exposure, but no differential expression was observed prior (48 h) or after (144 h) [22], which suggests that there is a narrow window in which transcriptomic regulation occurs. It has been proposed that bacterial endoparasites might need some time to penetrate the intestinal lining and colonize the gut epithelium of the *Daphnia* hosts before the host’s defense mechanisms are activated [4, 56].

### Late response

Exposure to parasites resulted in down-regulation of genes involved in chitin metabolism, as assessed 48 h after exposure. Parasites might be causing a weakening of the peritrophic matrix in the *Daphnia* gut. Chitin metabolism is a fundamental part of arthropod immunity [57], as the peritrophic matrix of arthropod guts forms a protective barrier against ingested pathogens [58, 59]. There are two types of chitin synthases, one responsible for the synthesis of cuticular chitin and the other associated with the gut peritrophic matrix [60, 61]. In our data, we observed a down-regulation of carbohydrate-binding module family 14 (CBM14), also known as peritrophin-A, which is a component of the peritrophic matrix of insect and animal chitinases [62, 63]. In the bumblebee - trypanosome gut parasite system, speritrophin and more genes associated with chitin metabolism were differentially expressed, suggesting an important role for the repair or restructuring of the peritrophic matrix in the host’s response to the parasite [64].

The down-regulation of genes related to c-type lectins at 48 h post exposure implies that *C. mesnili* could inhibit host cell recognition processes. C-type lectins are involved in the recognition of a variety of pathogens such as fungi, bacteria and viruses [65–67]. They bind carbohydrates, and mediate processes of cell adhesion, cell interactions and glycoprotein turnover [66]. In contrast to our finding of down-regulation of c-type lectins, a recent proteomic analysis of *D. magna* exposed to bacterium *P. ramosa* revealed their high abundance [68]. However, down-regulation of several c-type lectins was also observed in *D. pulex* exposed to the predatory phantom midge larvae *Chaoborus* [69]. Even if the detailed mechanisms of immune responses in *Daphnia* are still unknown, a threat from either predator or parasite appears to down-regulate c-type lectin expressions.

**Fig. 4:**
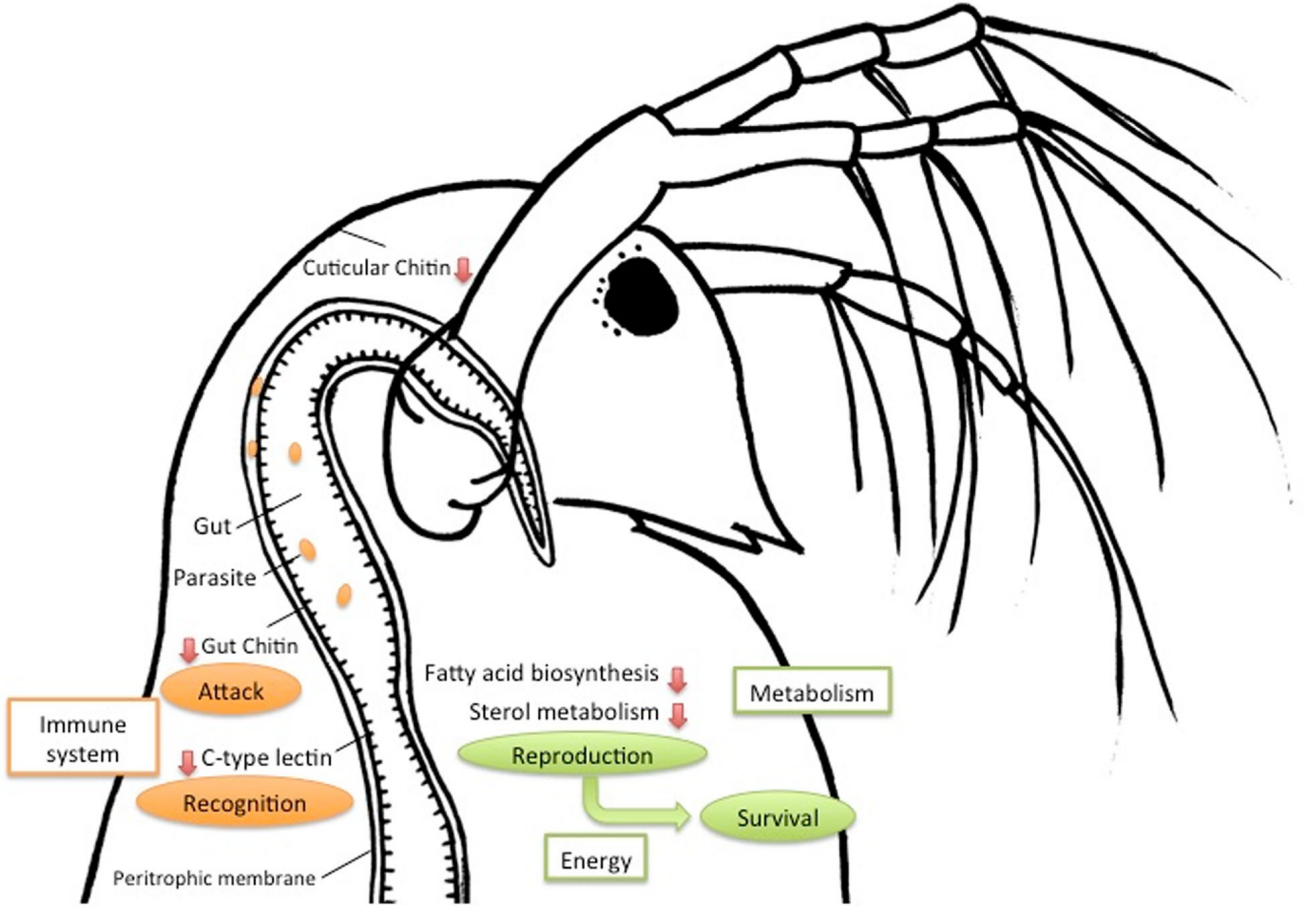
Schematic cartoon showing changes in immune system and lipid metabolism in *Daphnia galeata* exposed to gut parasite *Caullerya mesnili.* Parasite exposure results in down-regulation (red arrows) of immune pathways involved in recognition and attack. At the same time, general metabolic suppression indicates the energy use is shifted from reproduction to survival

Two plausible scenarios may explain the observed reduced expression of immune-related genes after parasite exposure (Fig. 4). 1) The parasites may evade host immunity, undermining host defenses for their development in the host’s gut. For example, the gut protozoan parasite *Crithidia bombi* modifies the immune response of its host bumblebee *Bombus terrestris* by down-regulating a large number of immune-related genes after infection [64]. Infection of the insect *Rhodnius prolixus* by the protozoan *Trypanosoma cruzi* reduces nitric oxide (NO) production which helps the parasite to complete its development in the digestive tract [70]. The principal pathways of innate immunity are conserved between arthropods and vertebrates [71, 72]. A marked down-regulation of immune-related genes was also reported from gilthead sea bream *Sparus aurata* infected by myxosporean enteric parasite *Enteromyxum leei*; immune suppressive responses were interpreted as a protection against host tissue damage by degradative enzymes [73, 74]. 2) Down-regulation of a number of metabolism-related genes provides an evidence of general metabolic suppression, including genes involved in lipid metabolism, fatty acid biosynthesis and immune pathways. Under stress conditions, many organisms shift the energy use from reproduction to survival or maintenance of homeostasis according to metabolic cost hypothesis [75–77]. Across a diverse array of insects, parasite infections drastically reduced host reproductive output and capacity [78]. This scenario is consistent with the experimental and field observations from *D. galeata* - *C. mesnili* system; infected host stops reproducing [28, 31]. Reduced or a completely shut down reproduction seems to be a typical response of *Daphnia* against various types of microparasites [4].

## Conclusions

We applied an advanced experimental system, the host *D. galeata* and its gut parasite *C. mesnili*, to examine the host’s responses on a trascriptomic level. The transcriptome profile of *D. galeata* after parasite exposure as well as the survey of the immune-related genes of *D. galeata* have yielded a new example of the parasites’ action to evade the immune defenses of the host, which is critical for disease spread and transmission.

## Additional files

Additional file 1: **Table S1.** Full list of *Daphnia galeata* transcripts that were up- or down-regulated at 4 h and 48 h after parasite exposure (based on *published* and de novo reference transcriptomes). (XLSX 82 kb)

Additional file 2: **Figure S1.** Venn diagram of the differentially up-regulated (↑) and down-regulated (↓) gene expression in *Daphnia galeata* based on the de novo reference transcriptome at 4 h (solid circle) and 48 h (dashed circle) after parasite exposure. (PNG 46 kb)

Additional file 3: **Table S2.** Annotation of all differentially expressed transcripts of *Daphnia galeata* (based on *published* and de novo reference transcriptomes). (XLSX 308 kb)

Additional file 4: **Table S3.** Gene ontology enrichment of down-regulated genes at 48 h after parasite exposure (based on the *published* reference transcriptome). (XLSX 60 kb)

Additional file 5: **Table S4.** A full list of the immune-related genes in the *published* reference transcriptome. (TXT 120 kb)
