# Supplementary figures and images for: Daphnia galeata responds to the exposure to an ichthyosporean gut parasite by down-regulation of immunity and lipid metabolism
Source: BMC Genomics. 2018 Dec 14;19:932. doi: 10.1186/s12864-018-5312-7 (PMC6295042; doi:10.1186/s12864-018-5312-7)

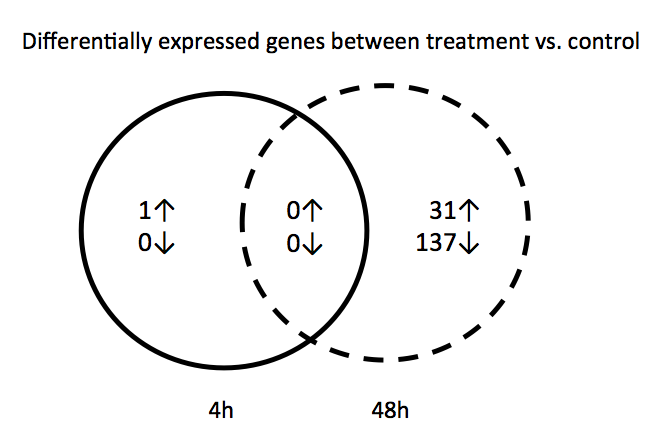

Supplement: Supplementary file 2 — Figure S1. Venn diagram of the differentially up-regulated (↑) and down-regulated (↓) gene expression in Daphnia galeata based on the de novo reference transcriptome at 4 h (solid circle) and 48 h (dashed circle) after parasite exposure. (PNG 46 kb) [file 12864_2018_5312_MOESM2_ESM.png]
